# The nsp15 Nuclease as a Good Target to Combat SARS-CoV-2: Mechanism of Action and Its Inactivation with FDA-Approved Drugs

**DOI:** 10.3390/microorganisms10020342

**Published:** 2022-02-01

**Authors:** Margarida Saramago, Vanessa G. Costa, Caio S. Souza, Cátia Bárria, Susana Domingues, Sandra C. Viegas, Diana Lousa, Cláudio M. Soares, Cecília M. Arraiano, Rute G. Matos

**Affiliations:** Instituto de Tecnologia Química e Biológica António Xavier, Universidade Nova de Lisboa, Avenida da Republica, 2780-157 Oeiras, Portugal; vanessa.costa@itqb.unl.pt (V.G.C.); cssouza@itqb.unl.pt (C.S.S.); catiabarria@itqb.unl.pt (C.B.); susanadomingues@itqb.unl.pt (S.D.); sviegas@itqb.unl.pt (S.C.V.); dlousa@itqb.unl.pt (D.L.); claudio@itqb.unl.pt (C.M.S.)

**Keywords:** SARS-CoV-2, ribonucleases, nsp15, inhibitors, FDA-approved compounds, therapeutics

## Abstract

The pandemic caused by SARS-CoV-2 is not over yet, despite all the efforts from the scientific community. Vaccination is a crucial weapon to fight this virus; however, we still urge the development of antivirals to reduce the severity and progression of the COVID-19 disease. For that, a deep understanding of the mechanisms involved in viral replication is necessary. nsp15 is an endoribonuclease critical for the degradation of viral polyuridine sequences that activate host immune sensors. This enzyme is known as one of the major interferon antagonists from SARS-CoV-2. In this work, a biochemical characterization of SARS-CoV-2 nsp15 was performed. We saw that nsp15 is active as a hexamer, and zinc can block its activity. The role of conserved residues from SARS-CoV-2 nsp15 was investigated, and N164 was found to be important for protein hexamerization and to contribute to the specificity to degrade uridines. Several chemical groups that impact the activity of this ribonuclease were also identified. Additionally, FDA-approved drugs with the capacity to inhibit the in vitro activity of nsp15 are reported in this work. This study is of utmost importance by adding highly valuable information that can be used for the development and rational design of therapeutic strategies.

## 1. Introduction

Severe Acute Respiratory Syndrome coronavirus 2 (SARS-CoV-2) emerged in 2019 in Wuhan, China [1] and rapidly spread worldwide, initiating a global pandemic. This virus has caused a major impact on health systems and in the world economy. In the last two years, scientists all around the world have extensively worked to better understand how this virus functions. A profound knowledge of the mechanisms involved in viral replication is of the utmost urgency and will surely contribute to the development of new treatments against COVID-19, the disease caused by SARS-CoV-2.

SARS-CoV-2 is a (+)-sense single-stranded RNA virus with a genomic RNA (gRNA) of ~30 kb in length [2] from the *Nidovirales* order. The gRNA, similar to what is observed in human RNA molecules, has a cap at the 5′-end and a poly(A) tail at the 3′-end. The presence of these traits allows the immediate translation of the viral genome in order to produce viral proteins, and prevent its degradation by intracellular ribonucleases (RNases) [3,4]. SARS-CoV-2 replication is a complex and crucial process. It involves the concerted action of viral and host proteins engaged in RNA polymerization, proofreading and capping of the RNA molecule essential in producing new viral proteins [5]. As an RNA virus, it requires a tight control on the production and processing of the RNA molecules. Ribonucleases are key proteins in this process [6]. The SARS-CoV-2 genome contains two large open reading frames (ORF), ORF1a and ORF1ab, with the latest coding for nonstructural proteins (nsp). Among the nsp proteins, we can find two ribonucleases, the exoribonuclease nsp14 and the endoribonuclease nsp15 [3]. Nsp14 has recently been characterized [7].

nsp15 is a conserved uridine-specific endoribonuclease only found in viruses from the order *Nidovirales* [8,9]. Considering that no homologs are present in any other RNA viruses, nsp15 is considered a genetic marker of this order of viruses [8,9]. Only distant homologs (XendoU) were identified in some prokaryotes and eukaryotes, which were shown to be involved in the processing of intron-encoded small nucleolar RNAs [10,11]. Nsp15 is a member of the nidoviral EndoU (also known as NendoU) family of proteins. It specifically cleaves RNA substrates endonucleolytically at the 3′ of pyrimidines, with a preference for uridine bases, releasing products with 2′ to 3′ cyclic phosphate and 5′-hydroxyl ends [12]. Nsp15 activity can be influenced by the secondary structure of the RNA, and has a preference for cleaving unpaired uridine bases in hairpin-structured RNAs [12]. It was also reported that the presence of 2′-*O*-ribose methyl groups on the RNA substrate can block the nsp15 activity [8]. Downstream to nsp15, the *nsp16* gene encodes for 2′-*O*-ribose methyltransferase [3]. Taking this into account, we can hypothesize that nsp16 may be a regulator of nsp15 activity by avoiding the cleavage of important viral RNA molecules during the infection process.

**Figure 1:**
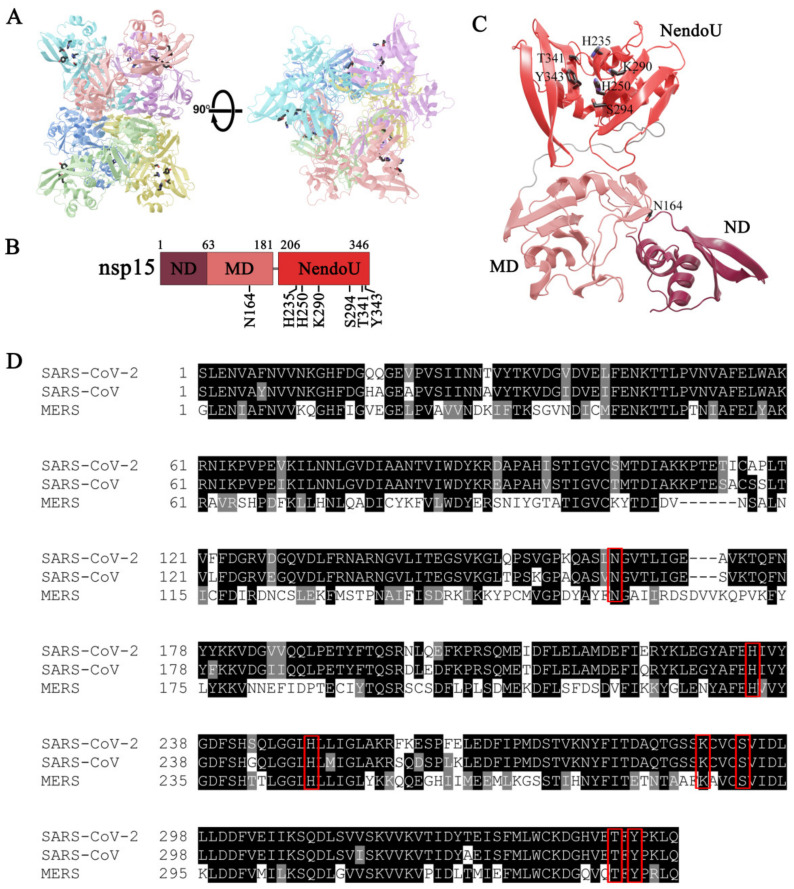
(**A**) Orthogonal views of the hexameric form of nsp15, which is formed by dimers of trimers. Monomers are colored in cyan, pink, violet, blue, green and yellow. Active site residues are shown as sticks. (**B**) nsp15 domain organization. ND, amino acids (aa) 1–63; MD, aa 64–181; NendoU, aa 206–346. The location of the conserved residues is represented. (**C**) Localization of the nsp15 domains in the monomer (PDB code 6W01). Mutated residues are shown as sticks. (**D**) Sequence alignment of nsp15 from SARS-CoV-2 (UniProt ID: P0DTD1), SARS-CoV (UniProt ID: P0C6X7) and MERS-CoV (YP_009047225). Residues are colored according to their conservation: in black, residues that are identical; in grey, residues that are not identical but have similar properties; in white, residues that are different. Residues mutated in this work are highlighted with a red box.

Biochemical studies performed with purified nsp15 from SARS-CoV and HCoV-229E revealed that this ribonuclease requires divalent ions as a cofactor to perform RNA cleavage, with a marked preference for Mn^2+^ [8,13]. These studies also revealed that Mn^2+^ influences nsp15 conformation, enhances RNA binding and, consequently, its NendoU activity [8,13]. Structural studies with nsp15 from different coronaviruses revealed that this protein is a hexamer formed by dimers of trimers, with a distinctive folding from other ribonucleases (Figure 1A) [14,15,16,17,18,19]. Each monomer is composed of three domains: N-terminal domain (ND), middle domain (MD) and the C-terminal catalytic NendoU domain (Figure 1B,C) [14,15,16,17,18,19]. The NDs are lined in the center of the hexamer, while the NendoU domains are facing the surface, which means that nsp15 has six active sites (Figure 1C) [14,15,16,17,18,19]. The stabilization of the hexamer is achieved through interactions of the ND, which contains the residues required for oligomerization, but the other domains also contribute to the oligomer interface [18,20]. The hexamer of nsp15 is the active form that exhibits the ability to bind RNA and the endonucleolytic activity [20]. The catalytic site at the NendoU domain is composed of the highly conserved residues H235, H250 and K290 (numbering according to the SARS-CoV-2 PDB 6VWW [17]) (Figure 1D) that constitute the catalytic triad, similar to what was described for bovine RNase A [21]. This suggests a similar mechanism of action, but while RNase A does not require a cofactor, nsp15 activity is stimulated by Mn^2+^ [12]. S294 and Y343 residues were postulated to interact with the substrate and confer a specificity for uridines [16]. The highly conserved T341 can also be involved in substrate recognition [14].

nsp15 plays an important role in coronavirus pathogenesis. It was reported that this RNase is responsible for the degradation of viral dsRNA intermediates, thus preventing host recognition [22,23]. By obstructing the recognition of the viral RNA, nsp15 directly contributes to delaying the type I interferon response. Several studies using live viruses in primary cells have confirmed that nsp15 acts as an interferon antagonist [22,23]. Later on, it was described that coronaviruses with mutations in the NendoU domain are less virulent, and their ability to activate the innate immune response is compromised [24]. For a review on this topic, please read Reference [25]. Taking all this into consideration, plus the fact that nsp15 is only found in nidoviruses with no close human homologs identified, this endoribonuclease is a very attractive target for developing new drugs to treat SARS-CoV-2 infections.

In this work, we elucidated the mechanism of action of nsp15 from SARS-CoV-2. Based on this knowledge, we also performed an *in silico* screening to find the best commercially available and approved drugs that might inhibit the NendoU activity of nsp15.

## 2. Materials and Methods

### 2.1. Plasmid Construction

Full-length nsp15 gene from SARS-CoV-2 (UniProt ID P0DTD1) was optimized for *Escherichia coli* expression and synthesized by GenScript (Piscataway, NJ, USA). The synthesized gene was subsequently cloned into the *Nde*I–*Bam*HI sites of commercial pET15b to generate pET15b_nsp15, which expresses an N-terminal His-tagged version of nsp15.

The single-point mutations N164A, H235A, H250A, K290A, S294A, T341A and Y343A and the double mutant T341A_Y342A were introduced into pET15b_nsp15 by overlapping PCR using the primers listed in Table S1. The double mutants S294A_T341A and S294_Y343A and the triple mutant S294A_T341A-Y343A were introduced into pET15b_nsp15_S294A also by overlapping PCR (Table S1). All the constructions were verified by sequencing at StabVida (Caparica, Portugal).

### 2.2. Protein Expression and Purification

Plasmids expressing nsp15 WT and mutant proteins were transformed into *E. coli* Rosetta cells (Novagen, Gibbstown, NJ, USA) for the expression of the recombinant proteins. Cells were grown in Terrific Broth medium supplemented with 150 μg/mL ampicillin and 50-μg/mL chloramphenicol at 30 °C to an OD_600_ of 0.5. At this point, protein expression was induced by addition of 0.5 mM IPTG, and bacteria were incubated for 4 h. Cells were pelleted by centrifugation and stored at −80 °C. Cell suspensions were lysed using the FastPrep-24 (MP Biomedical, Santa Ana, California, USA) at 6.5 m/s for 60 s in the presence of 0.5-mM PMSF. The crude extract was treated with Benzonase (Sigma-Aldrich, St. Louis, MO, USA) to degrade the nucleic acids, and clarified by a 30-min centrifugation at 10,000× *g*. Purification was performed in an ÄKTA FPLC™ system (Cytiva, Marlborough, MA, USA). The cleared lysate was subjected to a histidine affinity chromatography in a HisTrap HP column (Cytiva) equilibrated in Buffer A (40-mM Tris-HCl, pH 8, 150-mM NaCl and 10-mM imidazole). Proteins were eluted by a continuous imidazole gradient up to 500 mM in Buffer A. The fractions containing the purified protein were pooled together and concentrated by centrifugation at 4 °C with Amicon Ultra Centrifugal Filter Devices of 10000 MWCO (Millipore, Burlington, Massachusetts, EUA) and the buffer exchanged to Buffer B (20-mM Tris-HCl, pH 8). The proteins were then added to a HiTrap Q HP column (Cytiva) equilibrated in Buffer B to perform an anion exchange. Protein elution was achieved by a continuous NaCl gradient up to 1 M in Buffer B. Eluted proteins were concentrated by centrifugation at 4 °C with Amicon Ultra Centrifugal Filter Devices of 10000 MWCO (Millipore) and the buffer exchanged to Buffer C (20-mM Tris-HCl, pH 8 and 150-mM NaCl). Afterwards, the proteins were subjected to a size exclusion chromatography using a Superdex 200 Increase column (Cytiva) with a flow rate of 0.5 mL/min using buffer C. The samples collected were analyzed in a Novex™ 8–16% Tris-Glycine gel (Invitrogen™, Waltham, Massachusetts, EUA), followed by BlueSafe staining (NZYTech, Lisbon, Portugal). Samples with the highest purity were pooled together and concentrated by centrifugation at 4 °C with Amicon Ultra Centrifugal Filter Devices of 10000 MWCO (Millipore). All protein versions were purified at least twice in independent experiments to ensure reproducibility of the results. Proteins were quantified using the Bradford method, and 50% (*v/v*) glycerol was added to the final fractions prior storage at −20 °C. The purified proteins were analyzed by Western blot. One and a half micrograms of each protein was separated in a Novex™ 8–16% Tris-Glycine gel (Invitrogen™) and transferred to a nitrocellulose membrane (Hybond ECL, Cytiva) by electroblotting using the Trans-Blot SD semidry electrophoretic system (Bio-Rad, Hercules, CA, USA). Membranes were probed with a 1:10,000 dilution of anti-His antibody (Thermo Fisher Scientific, Waltham, MA, USA). ECL anti-mouse IgG conjugated horseradish peroxidase (Sigma-Aldrich, St. Louis, MO, USA) was used as the secondary reagent in a 1:10,000 dilution. Immunodetection was conducted via a chemiluminescence reaction using Amersham ECL Western Blotting Detection Reagents (Cytiva).

### 2.3. RNase Activity Assays

The synthetic 16-mer oligoribonucleotides R16.4 (5′-GAAGCGAAACCCUAAG-3′), 16comp (5′-AGUGGUUGGUGUCGGG-3′) and 30-mer (5′-CCCGACACCAACCACUAAAAAAAAAAAAAA-3′) (StabVida, Portugal) were used as substrates in the RNase activity assays. The RNA substrates were labeled at their 5′ end with [^32^P]-γ-ATP and T4 Polynucleotide Kinase (Ambion, Austin, TX, USA) in a standard reaction. MicroSpin G-50 columns (Cytiva) were used to remove excess [^32^P]-γ-ATP. In order to fold the R16.4 into its native structure, the RNA was resuspended in 10 mM of Tris-HCl, pH 8.0 and incubated 10 min at 80 °C, followed by 45 min at 37 °C. To obtain the double-stranded substrate 16–30 ds, the labeled 30-mer was hybridized to the unlabeled 16comp oligoribonucleotide. The hybridization was performed in a 1:5 (mol:mol) ratio by incubation for 10 min at 80 °C, followed by 45 min at 37 °C.

The activity assays were performed in a final volume of 12 µL containing an activity buffer with 25-mM HEPES, pH 7.4; 50-mM NaCl; 1-mM DTT; 10-mM NaHPO_4_ and 5 mM of either MgCl_2_, MnCl_2_, CaCl_2_, NiCl_2_, CuCl_2_, CoCl_2_ or ZnCl_2_, plus the protein nsp15 WT or respective mutants (protein concentrations are indicated in the figure legends). Some assays were performed with different concentrations of MnCl_2_ (1 mM, 5 mM, 10 mM, 15 mM or 20 mM) or with an activity buffer containing citrate or ammonium sulphate instead of sodium phosphate. A reaction was also done only in a HEPES buffer, without citrate, sulphate or NaHPO_4_ (as indicated in the figure legends). All the reactions were started by adding 50 nM of the RNA substrate and further incubated at 37 °C. Reaction controls were performed with all the components of the reaction without the enzymes. They were incubated in the same conditions as in the enzymatic reactions.

To test the drugs (Dutasteride, Etoposide, Golvatinib, Irinotecan, Meprednisone and Tasosartan), the compounds were previously incubated for 7 min at room temperature with nsp15 WT before the reactions were started (through the addition of RNA). All the drug compounds were solubilized in DMSO and serially diluted. Reaction controls were performed with the RNA substrates (without the enzymes), with either the activity buffer or buffer supplemented with DMSO. The DMSO concentration was kept constant, corresponding to the maximum amount of drugs tested in the assay. The control reactions were incubated in the same conditions.

For all the assays, aliquots of 4 µL were withdrawn at the time points indicated in the respective figures, and the reactions were stopped by adding a gel-loading buffer containing 80% *(v/v*) formamide and 10-mM EDTA. Reaction products were resolved in a 20% denaturant polyacrylamide gel (7-M urea). Signals were visualized by PhosphorImaging (TLA-5100 Series, Fuji, Cytiva). All the experiments were performed at least in triplicate for each independent purification. Disappearance of the substrate over time was quantified using ImageQuant software (Cytiva).

### 2.4. Sequence Alignment

nsp15 sequences from SARS-CoV-2 (UniProt ID: P0DTD1), SARS-CoV (UniProt ID: P0C6X7) and MERS-CoV (YP_009047226) were aligned using T-coffee [26]. The final figure presented was built with Boxshade (https://embnet.vital-it.ch/software/BOX_form.html, accessed on 3 September 2021). Sequence identity and similarity were determined in https://www.bioinformatics.org/sms2/ident_sim.html, accessed on 3 September 2021).

### 2.5. Docking Calculations

The tridimensional structures of the target molecules were obtained from the Approved, Investigational and Experimental subsets of the Drugbank database [27]. All molecules were prepared using AutoDockTools [28] by adding Gasteiger charges and AutoDock4 atom types. Flexible bonds were allowed to rotate, nonpolar hydrogens were merged and missing polar hydrogens were added. The structure present in PDB ID 6WXC was chosen to be the receptor, since this protein was co-crystalized with a drug and a phosphate ion bound together to the active site. The protein was prepared with AutoDockTools in a similar way as the drugs, and all atoms that were not part of the protein or the phosphate ion were removed. Docking calculations were performed with the program AutoDock Vina [29] in a searching box with sizes 22 Å, 24 Å and 20 Å (*x*-, *y*- and *z*-axis, respectively) centered at the nsp15 active site. The exhaustiveness parameter was set to 20 for all runs, and only the poses with the lowest binding energies were considered for further analysis.

## 3. Results

### 3.1. nsp15 Endoribonuclease Is Active as a Hexamer

**Figure 2:**
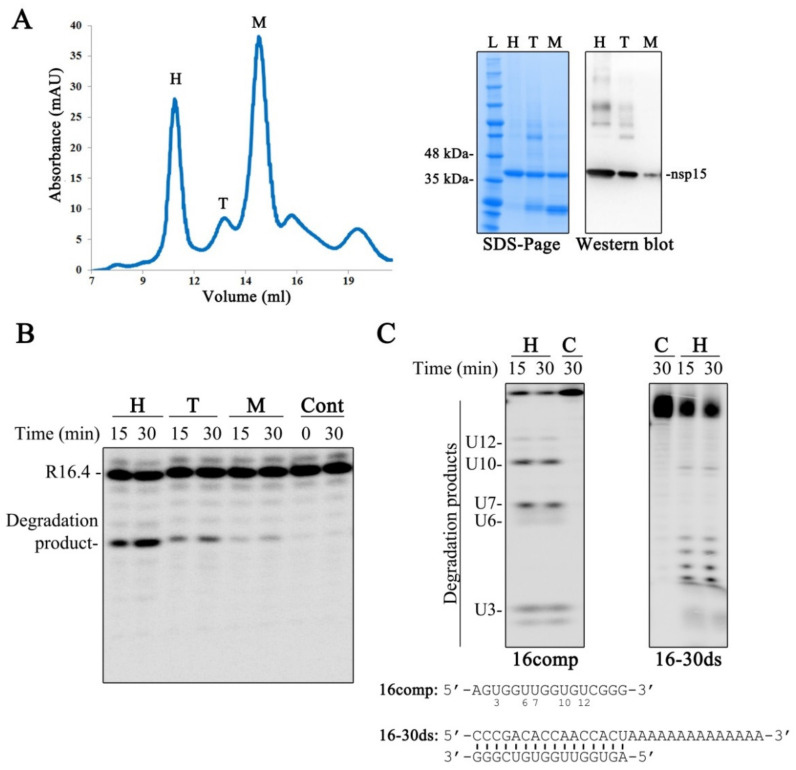
Characterization of SARS-CoV-2 nsp15. (**A**) Left panel: chromatogram obtained during nsp15 size exclusion chromatography; right panels: SDS-PAGE gel with the purified fractions collected during the SEC and the respective Western blot. M, monomer; T, trimer; H, hexamer; L, Protein ladder. (**B**) In vitro activity of the purified fractions H, T and M using 50 nM of R16.4 RNA substrate and 500 nM of protein. (**C**) In vitro activity of nsp15 in the hexamer conformation (500 nM) in the presence of a single-stranded RNA (16comp) and a double-stranded RNA (16–30 ds), which are schematically represented in the bottom. Reactions were analyzed on 7-M urea/20% polyacrylamide gels. C, control reactions; time points are indicated in the top of each panel. All the experiments were performed at least in triplicate.

We purified the SARS-CoV-2 nsp15 recombinant protein. As presented in Figure 2A, the chromatogram obtained during the size exclusion chromatography step shows the presence of three main protein peaks that elute between 9 and 16 mL of column volume. It was already demonstrated that nsp15 is a hexamer formed by dimers of trimers [14,15,16,17,18,19]. As such, and taking into consideration the chromatograms with standard components, we postulate that the first peak corresponds to the hexamer (H), the second and smaller peak to the trimer (T) and the last one to the monomer (M) (Figure 2A). The fractions collected for each peak were analyzed in an SDS-PAGE gel. We confirmed the presence of a protein that migrates at 41 kDa (Figure 2A), which is consistent with the expected molecular weight of the recombinant His-tagged nsp15. It is possible to observe bands with higher molecular weight in the gel, namely in the fractions that correspond to the hexameric and trimeric forms. By Western blot, using antibodies specific for the His-tag tail of the protein, we confirmed that those bands correspond to nsp15 oligomers, which are not present in the fraction of the monomeric form (Figure 2A). We could also observe the presence of bands with lower molecular weights in the trimer and monomer fractions (Figure 2A). These bands most likely corresponded to nsp15 that was degraded during the purification process.

We tested the endonucleolytic activity of the purified fractions, using the synthetic RNA substrate R16.4, previously reported in Bhardwaj et al. [12]. This substrate is a 16-nucleotide (nt)-long RNA that contains only one uridine, rendering an unique cleavage event by nsp15 [12]. The results in Figure 2B show that nsp15 is more active as a hexamer. It retains some activity in the trimeric form, and only residual cleavage is observed in the monomeric form (Figure 2B). These results are in agreement with previous studies [20]. Moreover, it is possible to observe that the incubation of SARS-CoV-2 nsp15 with the R16.4 RNA substrate originates a single degradation product, due to cleavage on the U residue (Figure 2B), which demonstrates the preference of this enzyme for uridines. We also tested the activity of nsp15 over the ssRNA 16comp that presents five uridines in its sequence (Figure 2C). As expected, it is possible to observe the generation of five degradation products that correspond to the cleavage at the uridines (Figure 2C, left panel), reinforcing the marked preference of nsp15 for U residues. It was recently described that nsp15 has a preference for purines at the 3′ position of the cleaved uridines [30]. Interestingly, the 16comp RNA has purines in the 3′ position of the first four uridines (U3, U6, U7 and U10) counting from the 5′ end, whereas the last uridine (U12) is followed by a pyrimidine residue (Figure 2C). Analyzing the degradation pattern obtained, we can observe that cleavage is much less efficient at U12 (Figure 2C, left panel), confirming that SARS-CoV-2 nsp15 prefers purines 3′ to uridines.

It was already reported that nsp15 can cleave both ssRNA and dsRNA [8,13]. Therefore, we also tested SARS-CoV-2 nsp15 activity using the 16–30 ds RNA molecule. Our results show that this endoribonuclease is able to cleave this substrate (Figure 2C, right panel), confirming that SARS-CoV-2 nsp15 can cleave both ss and dsRNA molecules.

### 3.2. nsp15 Activity Is Stimulated in Different Buffer Conditions, with a Marked Preference for Manganese

**Figure 3:**
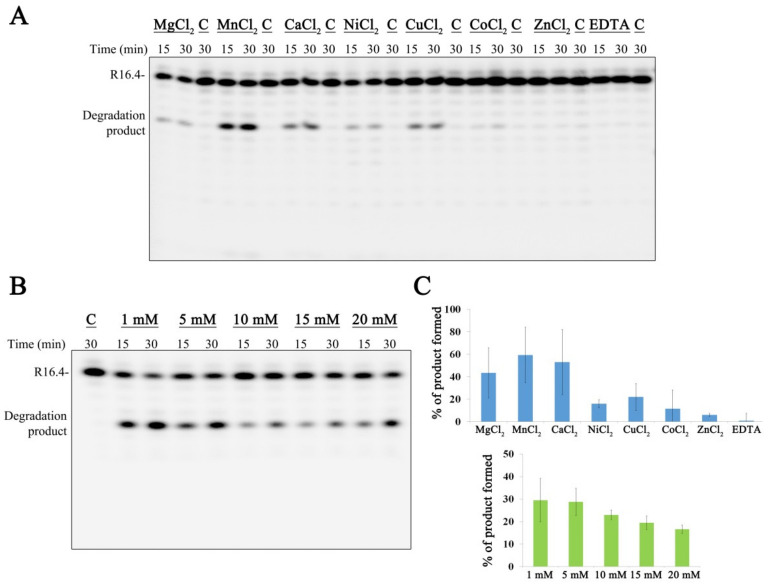
Effect of divalent ions in SARS-CoV-2 nsp15. (**A**) Five hundred nM of nsp15 were incubated with 50 nM of R16.4 RNA substrate in the presence of 10 mM of different divalent ions. (**B**) Five hundred nM of nsp15 were incubated with 50 nM of R16.4 RNA substrate in the presence of different MnCl_2_ concentrations (1–20 mM). Reactions were analyzed on 7 M urea/20% polyacrylamide gels. C, control reactions; time points are indicated in the top of each panel. All the experiments were performed at least in triplicate. (**C**) Quantification of nsp15 protein activity (% of RNA degradation product generated) in the presence of different divalent ions (on the top) and in a growing range of different MnCl_2_ concentrations (on the bottom).

It was already reported that the activity of nsp15 is highly enhanced in the presence of manganese [13,15,16]. In order to assess the role of other divalent ions over SARS-CoV-2 nsp15 activity, we tested the activity of the enzyme in the presence of Mg^2+^, Mn^2+^, Ca^2+^, Ni^2+^, Cu^2+^, Co^2+^ and Zn^2+^ (Figure 3A). Our results clearly illustrated that SARS-CoV-2 nsp15 presents maximum activity in the presence of Mn^2+^ (Figure 3A,C). Interestingly, Ca^2+^ and Cu^2+^ can also stimulate nsp15 activity. In the presence of Ni^2+^ and Co^2+^, the enzyme retains residual activity, while Zn^2+^ does not influence the activity of this RNase (Figure 3A,C). No degradation was observed in presence of the chelating agent EDTA in these conditions. We also tested if the presence of Mn^2+^ or Mg^2+^ would influence nsp15 oligomerization, which would help explain the increase in nsp15 activity. However, the chromatograms were similar to the one obtained in the absence of divalent ions that is presented in Figure 2A. Altogether, these results confirm the importance of divalent ions, namely Mn^2+^, for the activity of nsp15 from SARS-CoV-2, similar to what was described for other counterparts [13,15,16], and exclude the involvement of divalent ions in oligomerization. Studies performed with MERS-CoV and SARS-CoV nsp15 have shown that the presence of Mn^2+^, but not Mg^2+^, increases the affinity to the RNA molecule [12,16]. This suggests that Mn^2+^ stimulates nsp15 activity by increasing the ability to bind to RNA substrates and not by binding to its active site. In fact, nsp15 structure suggests that divalent ions may stabilize the protein [18,31]. Next, we tested the nsp15 activity using different Mn^2+^ concentrations, from 1 to 20 mM. The results obtained showed that lower Mn^2+^ concentrations are more effective in stimulating nsp15 activity, with the protein presenting a higher ability to cleave RNA in the presence of 1 mM of Mn^2+^ (Figure 3B,C).

**Figure 4:**
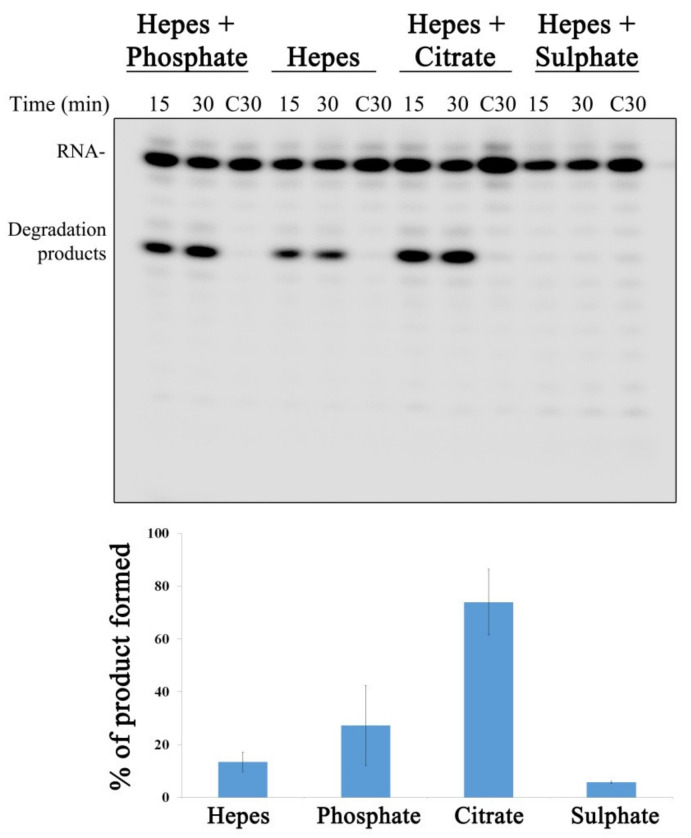
The influence of different buffer compositions in SARS-CoV-2 nsp15 activity. On the top, 500 nM of nsp15 were incubated with 50 nM of R16.4 RNA substrate in the presence of buffers with different compositions, as indicated on the top of each panel. Reactions were analyzed on 7 M urea/20% polyacrylamide gels. C, control reactions; time points are indicated in the top of each panel. All the experiments were performed at least in triplicate. On the bottom, quantification of nsp15 protein activity (% of RNA degradation product generated) in the presence of buffers with different compositions.

Kim et al. reported that nsp15 was crystallized in the presence of a citrate, which was bound to the protein active site [18]. The citrate was shown to make hydrogen bonds with several key and highly conserved residues found in the nsp15 active site, including H235, H250, K290 and T341 (highlighted in Figure 1D). Moreover, it also forms Van der Waals contacts with Tyr343. All these interactions were postulated to stabilize the residues from the active site, thus ordering the NendoU domain [18]. In another study where the nsp15 structure bound to nucleotides was solved, it was possible to observe the presence of a phosphate ion bound to the active site that also interacted with H235, H250, T341 and K290 residues [31]. It was described that the nsp15 active site is structurally similar to that of RNase A. Sulfate was found to inhibit the ribonucleolytic activity of RNase A [32]. Considering the similarities between both ribonucleases, sulfate could also influence nsp15 activity. In order to determine the role of citrate, phosphate and sulfate over nsp15 activity, we ran activity assays in the presence of these compounds. Our results showed that, in the conditions tested, both phosphate and citrate enhanced nsp15 activity (Figure 4). In contrast, in the presence of sulfate, nsp15 activity was completely blocked (Figure 4). These results indicate that the activity of nsp15 from SARS-CoV-2 is highly influenced by the presence of different chemical groups in the reaction, which can be valuable information when designing compounds to inhibit nsp15 activity.

### 3.3. A Residue from ND from SARS-CoV-2 nsp15 Is Important for Hexamer Formation While Residues in NendoU Are Crucial for the Activity of the Protein

**Figure 5:**
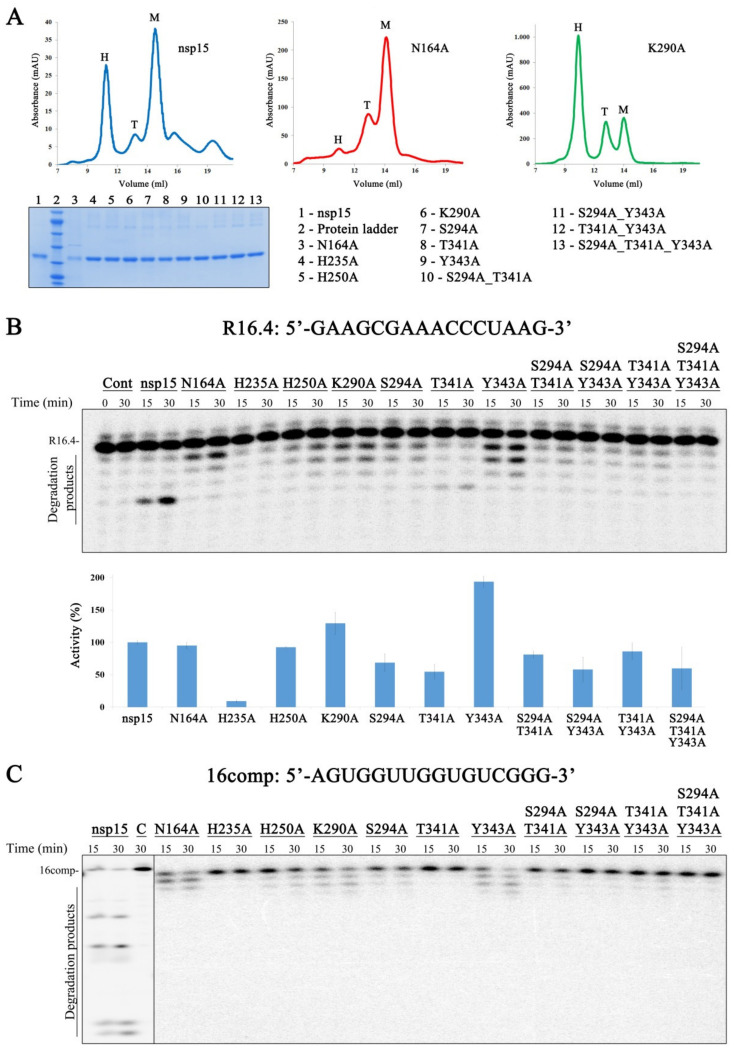
Effect of nsp15 mutations on its endonucleolytic activity. (**A**) On the top, chromatograms obtained during nsp15 wt, N164A and K290A size exclusion chromatographies; M, monomer; T, trimer; H, hexamer; on the bottom, a SDS-PAGE gel with all hexameric fractions of the purified proteins. (**B**) On the top, 800 nM of either nsp15 wt or nsp15 mutant versions were incubated with 50 nM of R16.4 RNA substrate; on the bottom, quantification of nsp15 wt and mutants activity in the presence of R16.4 RNA (% of RNA substrate degraded). The activity of nsp15 wt was considered as 100%. (**C**) 800 nM of nsp15 wt or each mutant version were incubated with 50 nM of 16comp RNA substrate. Reactions were analyzed on 7 M urea/20% polyacrylamide gels. C, control reactions; time points are indicated in the top of each panel. All the experiments were performed at least in triplicate.

nsp15 from different coronaviruses presents a high degree of sequence identity and similarity. SARS-CoV-2 nsp15 shares 88.7% sequence identity and 94.8% of similarity with the SARS-CoV homolog, and 50.1% sequence identity and 63.9% similarity with the homolog from MERS-CoV. The nsp15 active site is constituted by three highly conserved residues (H235, H250 and K290) (Figure 1D) that were postulated to be important for the activity of the enzyme [14,16,18]. Moreover, residues S294, T341 and Y343 were described as important for uridine specificity [16,24,33]. Studies conducted with nsp15 from MERS-CoV have shown that amino acids from the N-terminal portion of the protein (N38, Y58 and N157 in MERS-CoV nsp15 protein) are crucial for hexamer formation [16]. From these, N164 (corresponding to N157 in MERS-CoV) is the only one found in SARS-CoV-2 nsp15 (Figure 1D). Considering the conservation of these amino acids among the three coronaviruses (Figure 1D), and in order to address their role in the activity of SARS-CoV-2 nsp15, we constructed the N164A, H235A, H250A, K290A, S294A, T341A and Y343A point mutants; the S294A_T341A, S294_Y343A and T341A_Y342A double mutants, and the triple mutant S294A_T341A_Y343A. All the mutants were expressed and purified in the same conditions as the wild-type. Size exclusion chromatography showed that the majority of the purified mutants had an elution profile similar to that of the wild-type nsp15, with most of the proteins in hexameric and monomeric forms (Supplementary Figure S1). Interestingly, most of the N164A mutant protein was found in a monomeric form, and only a small peak was detected in the hexameric form (Figure 5A, middle panel). This result showed that N164 residue, which is located in the interface between the N-terminal and NendoU portions of adjacent monomers of nsp15, is crucial for the formation of stable hexamers. This is in agreement with studies performed with MERS-CoV nsp15, where the corresponding residue was also shown to be critical for the formation of the hexamer [16]. In contrast, the K290A mutant presents most of the proteins in the hexameric form (Figure 5A, right panel). This indicates that this residue, which is in the interface between monomers but whose side chain is located in the active site of nsp15, plays a role in the formation of the hexamer. The same behavior was already described for the corresponding residue in SARS-CoV [20]. Taking into consideration that the hexamer is the active form, we analyzed the activity of the fractions corresponding to the hexameric form of all the mutants. For this, we concentrated the samples of each peak corresponding to the hexamer (in order to have enough protein for the activity assays, especially for the N164A mutant) and used two different RNA substrates, R16.4 and the 16comp. As already described above, SARS-CoV-2 nsp15 only cleaves the R16.4 substrate in one place, rendering it a single degradation product (Figure 2B). In contrast, the 16comp substrate has five uridines in its composition, which originates five different degradation products (Figure 2C). We observed that the nsp15 N164A mutant degrades both RNA substrates near the 3′ end and not at the uridine bases as the wild-type protein (Figure 5B,C). This may suggest that mutations in the N-terminal region of the protein may affect its overall folding, compromising the integrity of the active site (NendoU domain). A folding effect is further supported by the unlikelihood of N164 being part of the important interactions between monomers. In fact, the only favorable interaction between N164 and the adjacent monomers are hydrogen bonds with T282. Furthermore, the mutation N164A would represent a decrease of less than 5% in the total contact surface area between monomers. Taking this into account, it is more likely that the predominance of the monomeric form was caused by folding problems and not by the direct absence of hexamer-stabilizing interactions. Although the formation of the hexamer has been severely compromised, N164A still retains a similar activity to the wild-type in terms of substrate consumption (Figure 5B).

nsp15 His235, His250 and Lys290 residues constitute the catalytic triad. Mutating these residues into alanines rendered different results, indicating that they play distinct roles in catalysis. The H235A mutant is almost inactive, while H250A and K290A still retain unspecific activity, similarly to what was observed for the N164A mutant (Figure 5B,C). These results contrast with the ones obtained with MERS-CoV and SARS-CoV nsp15, where changing these residues into alanine rendered a protein with no detectable activity [16,20]. It was already postulated that S294, T341 and Y343 are important for the uridine specificity demonstrated by nsp15 [16,33]. In fact, S294A and Y343A mutants present an unspecific pattern of degradation for both RNA substrates (Figure 5B,C). By quantifying the disappearance of the substrate, we verified that S294A retains ~70% of the activity when compared to the wild-type, whereas the Y343A mutant has twice more activity (Figure 5B). These results confirm that residues S294 and Y343 are, in fact, important for uridine specificity but not for the ability of nsp15 to cleave RNA. Although T341 was also postulated to be involved in this preference of cleavage, our results show that the nsp15 T341A mutant version maintains the specificity for U residues (Figure 5B). However, this substitution affects the ability of nsp15 to efficiently cleave RNA substrates, rendering a protein with half of the activity when compared to the wild-type (Figure 5B). The double mutants (S294A_T341A, T341A_Y343 and S294A_Y343A) exhibited reduced activity and a similar degradation pattern to that observed in the corresponding single mutants, while the triple mutant (S294_T341A_Y343) presented residual activity (Figure 5B,C). These results confirm the importance of these amino acids for nsp15 activity. Altogether, our biochemical analysis points to the importance of several conserved amino acids for nsp15 activity, sequence specificity and oligomerization.

This information is highly valuable to discover compounds that may affect NendoU activity that can be used for the treatment of viral infections provoked by a coronavirus.

### 3.4. Searching for SARS-CoV-2 nsp15 Inhibitors

**Figure 6:**
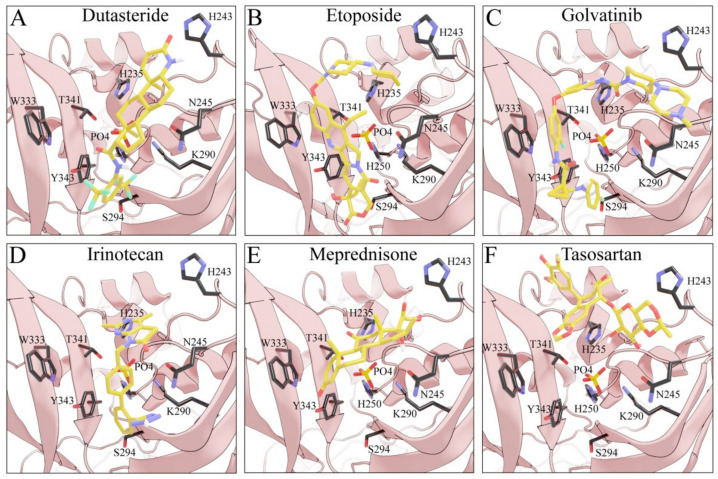
Docking solutions for the six selected drugs (**A**) Dutasteride, (**B**) Etoposide, (**C**) Golvatinib, (**D**) Irinotecan, (**E**) Meprednisone and (**F**) Tasosartan are shown with the carbon atoms in yellow. Key residues of the nsp15 active site are depicted with the carbon atoms in black. The active site and the phosphate ion (PO_4_) are covered by Dutasteride, Irinotecan, Golvatinib and Tasosartan. Meprednisone and Etoposide bind only to the top portion of the nsp15 active site.

Considering the important role that nsp15 plays in coronavirus pathogenesis, we performed a search for predicted drug candidates that could block the activity of this crucial viral enzyme. We conducted a virtual screening to find inhibitors of nsp15 among 8346 drugs from the Drugbank database that are either approved for clinical use or that are under investigation [27]. Given that the nsp15 activity is increased when incubated in a phosphate-containing buffer, and based on the findings of Kim et al. [31] where nsp15 was co-crystalized with a repurposed drug together with a phosphate ion in the active site, we decided to perform the docking calculations in the presence of a phosphate ion. The drugs with the lowest binding energies from our screening were compared with the ones found by Xu et al. [34]. Six drugs were chosen for further analysis, namely Dutasteride, Irinotecan, Meprednisone, Etoposide, Golvatinib and Tasosartan. The binding affinities ranged from −7.5 kcal/mol up to −9.2 kcal/mol (Table 1). Two binding patterns were observed among the selected drugs. Dutasteride, Golvatinib, Irinotecan and Tasosartan were flexible and long enough to cover the phosphate ion inside the active site (Figure 6A,C,D,F). The extremities of these molecules were located in two regions: one near H235 and another near Y343 and S294. Dutasteride and Golvatinib have fluorinated groups that interact with S294. Together with these halogenated groups, there are aromatic rings that may form pi-stacking interactions with Y343 (Figure 6A,C). Both S294 and Y343 were shown in this study to be important for uridine specificity. Golvatinib is further stabilized by a pi-stacking interaction between another fluorinated aromatic ring with W333. Taken together, these intermolecular interactions may account for the high binding affinity predicted for Golvatinib. Tasosartan also has an aromatic ring near Y343, but it is not well-aligned to form pi-stacking. No specific strong interactions were formed in the case of Irinotecan. Contrary to the first group of molecules, Etoposide and Meprednisone were located only at the top of the active site, interacting with H235 and partially interacting with the phosphate ion (Figure 6B,E). These two molecules did not have specific interactions with nsp15 and were largely exposed to the solvent, which may explain their low binding affinities. The binding modes observed for the selected drugs are compatible with a scenario of competitive inhibition of nsp15 by occupying the space where the RNA substrate would be bound around the active site.

**Figure 7:**
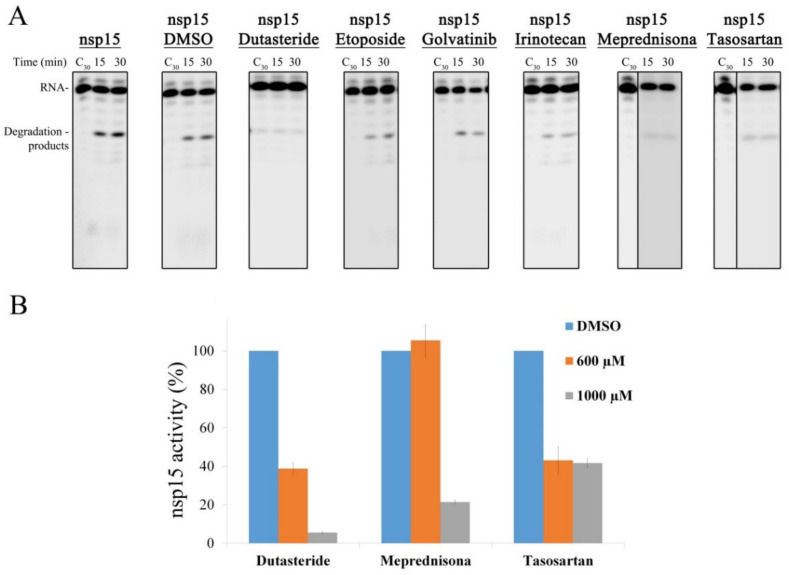
Effect of different drugs in SARS-CoV-2 nsp15 activity. (**A**) 500 nM of nsp15 were incubated with 50 nM of R16.4 RNA substrate in the presence of 1 mM of each drug indicated on the top of the respective panel. Reactions were analyzed on 7-M urea/20% polyacrylamide gels. C, control reactions; time points are indicated in the top of each panel. All the experiments were performed at least in triplicate. (**B**) Quantification of nsp15 activity in the presence of different concentrations of Dutasteride, Meprednisone and Tasosartan. The activity of nsp15 in the presence of DMSO was considered 100%.

The selected compounds were tested regarding their ability to inhibit nsp15 activity. In vitro RNA cleavage assays were performed using the R16.4 RNA substrate in the presence or absence of the selected compounds. The RNA and nsp15 concentrations were kept constant. Reaction controls were performed in the absence of the enzymes, with a buffer supplemented with DMSO, since this solvent was used to solubilize all the chemical compounds. To evaluate the biochemical inhibitory effect of the drugs over SARS-CoV-2 nsp15, 1000 µM of each compound were tested. Etoposide, Golvatinib and Irinotecan seemed not to significantly influence the activity of the enzyme in the conditions tested (Figure 7A). However, Dutasteride, Meprednisone and Tasosartan showed ability to inhibit the endoribonuclease activity of nsp15 (Figure 7A). Considering the inibitory effect of these last drugs, we quantified the activity of nsp15 protein in the presence of 600 and 1000 µM of each of the compounds (Figure 7B). In the presence of 600 µM of Dutasteride or Tasosartan, nsp15 retained only ~40% of its activity, while the same concentration of Meprednisone had no effect on the nsp15 activity (Figure 7B). Increasing the concentration of Dutasteride to 1000 µM caused a drastic effect on nsp15 activity, since it almost abolished the ability of the enzyme to cleave the RNA substrate (Figure 7B). 1000 µM of Meprednisone considerably reduced nsp15 activity (Figure 7B). For Tasosartan, the increase in drug concentration had no effect, since the activity was similar to the one detected in the presence of 600 µM (Figure 7B). These results confirmed that nsp15 activity is blocked or significantly reduced in the presence of Dutasteride, Meprednisone and Tasosartan, being Dutasteride the most effective one. These experiments provided important insight into chemical compounds that may be further developed and tested for COVID-19 treatment.

## 4. Discussion

nsp15 is an uridylate-specific endoribonuclease that is highly conserved and present in all coronaviruses [8,11,13,18,35]. This RNase plays a critical role in CoV replication and host immune system evasion, which makes it a very attractive target for drug design [22,36].

In this work, we performed the biochemical characterization of SARS-CoV-2 nsp15 and confirmed its endoribonucleolytic activity [8,13,18,19,30,31,37]. Additionally, we showed different contributions of divalent metal ions for SARS-CoV-2 nsp15 endoribonucleolytic activity. The enzyme showed a preference for Mn^2+^ over Mg^2+^, Ca^2+^ and Cu^2+^, while Ni^2+^ and Co^2+^ supported residual activity, and Zn^2+^ inhibited the catalysis. We previously reported that the RNase activity of SARS-CoV-2 nsp14 is also affected by the presence of different metal cofactors [7]. Additionally, Viswanathan et al. described the dependence and preference of SARS-CoV-2 2′-*O*-methyltransferase nsp16 for distinct divalent metal ions [38]. The three enzymes nsp14, nsp15 and nsp16 have a crucial role in innate immune system evasion [22,39,40]. It is tempting to speculate that the surrounding environment could influence the viral response to the host immune system through the modulation of protein activity. Interestingly, Zn^2+^ was shown to inhibit SARS-CoV replication in vitro [41], which is in agreement with our results that demonstrated the inhibition of SARS-CoV-2 nsp15 activity by Zn^2+^. Moreover, different concentrations of metal ions in blood of patients have been associated with severe illness and the fatal outcome of COVID-19 [42]. This evidence opens the door to explore the biological role of metals in the course of SARS-CoV-2 infection.

nsp15 activity and cleavage specificity have been shown to play important roles in coronavirus life cycles. Our results demonstrated the ability of SARS-CoV-2 nsp15 to cleave single- and double-stranded RNA substrates, with a marked preference for U residues. During CoV replication, the generation of dsRNA intermediates and the 5′-polyuridine (PUN) RNAs produced at the 5′ end of the negative strand are known to trigger type I interferon (IFN-I) [24,43,44]. The endoribonucleolytic activity of nsp15 was found to destroy these intermediate RNAs, hindering the activation of the host innate immune response [22,23,25]. Indeed, nsp15 has been reported as one of the major interferon antagonists from SARS-CoV-2. Thus, it plays a very important role in viral RNA replication and pathogenesis [22,23,25]. Indeed, Nguyen et al. [45] observed that the deletion or inactivation of nsp15 significantly reduced SARS-CoV-2 genome replication. Additionally, in the human Coronavirus HCoV-229E, a nsp15 mutant was found to abolish viral RNA synthesis [8]. In murine hepatitis virus (MHV), nsp15 mutant viruses were defective for viral infection, and deleting parts of the equine arteritis virus (EAV) counterpart rendered a nonviable virus [46,47].

It is known that the biological unit of coronavirus nsp15 homologs is a hexamer, which is crucial for enzymatic activity and RNA binding [16,18,19,20]. Here, we show that oligomerization is critical for SARS-CoV-2 nsp15 enzymatic activity, being the hexamer of the active form. We also found that the residue N164 is important for nsp15 oligomerization but still retains the ability to cleave RNA; moreover, the oligomeric state seems to impact the U discrimination in SARS-CoV-2 nsp15. In fact, the nsp15 hexamer might be necessary for the stability and organization of the catalytic site and engagement of the RNA molecule. This is the first time that nsp15 oligomerization has been related with its cleavage specificity. Indeed, impacting the efficiency of the enzyme to cleave uridines could be an effective approach to affect viral pathogenesis, since it might have serious consequences in terms of host innate evasion. We also demonstrated that substitution of the residues from the catalytic triad have different outcomes, suggesting a more important role of H235 in catalysis, while the H250A and K290A mutants still retain unspecific activity. This was not observed in the SARS-CoV and MERS counterparts, where these mutations abrogated the activity of nsp15 [16,20]. Thus, our work revealed differences on the functionality of the enzyme that might be related to SARS-CoV-2 pathogenesis. We also demonstrated that the S294 and Y343 residues, which were postulated as responsible for uridine discrimination [16], are, in fact, important for the ability of nsp15 to recognize and cleave U residues but are not critical for the ability of the protein to cleave RNA, as previously described [16,24,33]. Our results indicated that attacking critical nsp15 residues, alone or in combination, could result in an inactive enzyme and, as consequence, a decreased SARS-CoV-2 infectivity. Supporting this, a SARS-CoV-2 replicon system (developed to avoid working with infectious SARS-CoV-2 virus) with a nsp15 H235A_H250A double mutation was shown to have its replication ability highly impaired [45].

Viral infections are known to dramatically alter the host cell metabolism for its replication and spread. SARS-CoV-2 infection was reported to change the metabolic profile in cell lines and lung air–liquid interface cultures [48]. Thus, understanding the influence of metabolites in the course of a viral infection would be extremely valuable. Citrate is a metabolite that plays a key role in pro- and anti-inflammatory homeostasis, contributing to both the antiviral immune response and virus-induced inflammation [49]. In this work, we found that citrate enhances the activity of SARS-CoV-2 nsp15. This finding opens a link between the host cell metabolism and viral RNA degradation pathways. Interestingly, this metabolite was found to co-crystalize with SARS-CoV-2 nsp15 [18]. Citrate binds to the protein active site, promoting its stabilization through Van der Waals contacts with Y343 and hydrogen bonds with the residues H235, H250, K290 and T341 [18], explaining the increase of the endoribonucleolytic activity here observed. It is not the first time that citrate has been reported to modulate the activity of an RNase. The activity of the phosphorolytic ribonuclease PNPase has previously been shown to be inhibited by citrate in the bacteria *E. coli* [50] and *Campylobacter jejuni* (Matos et al., unpublished results). Recently, a structure of SARS-CoV-2 nsp15 bound to Tipiracil was solved, and a phosphate ion was detected in the catalytic pocket [31], which led us to investigate the influence of phosphate over nsp15 activity. Our results demonstrated that phosphate also increases the NendoU activity of SARS-CoV-2 nsp15. It was already described that more than half of the known proteins interact with phosphate [51]. Additionally, the metabolism of phosphate was suggested to play a critical role in SARS-CoV-2 pathogenesis. For instance, SARS-CoV-2 can induce the depletion of cellular ATP, which consequently interferes with IFN-1 production and induces a cytokine storm [52]. We also discovered that sulfate is capable of inhibiting the endonucleolytic activity of SARS-CoV-2 nsp15. Sulfate ions mimic the phosphate group of RNA and have been used to map enzyme-binding sites [53]. The activity of RNase A, whose active site is structurally similar to that of nsp15, was also found to be inhibited by sulfate [32]. This finding is a significant breakthrough that can be used to rationally design drugs to inhibit nsp15 activity.

Despite the extensive research, the new possible therapies and the already existing vaccines, the worldwide response to the COVID-19 pandemics is still insufficient, and we are lagging behind the virus. In this manuscript, we performed a virtual screening of drugs that could bind the nsp15 active site and block its activity. From the results obtained, we selected the best six drugs to perform in vitro activity assays. Our results demonstrated that Meprednisone, Tasosartan and Dutasteride significantly reduced nsp15 activity, Dutasteride being the most effective compound. Many empirical therapeutic options have been used to treat COVID-19 patients based on the disease progression. Patients with severe COVID-19 can develop a systemic inflammatory response that can lead to lung injury and multisystem organ dysfunction. There are indications that corticosteroids (such as dexamethasone) and other immunosuppressive agents might prevent or mitigate these deleterious effects [54]. Meprednisone is a glucocorticoid and a methylated derivative of prednisone. Prednisone is converted by liver enzymes to the active form prednisolone. Prednisolone is already used to treat a wide range of health problems, such as allergies, blood disorders, skin diseases, infections, cancer, etc. A smaller randomized controlled trial evaluated the effect of methylprednisolone in patients with COVID-19. Although the trial had a low sample size, it showed that the methylated form of prednisolone reduces mortality in hospitalized patients older than 60 years with COVID-19 [55]. Our observation that meprednisone (the methylated and nonmetabolized form of prednisone) has the ability to inhibit the activity of SARS-CoV-2 nsp15 is a good indicator that this drug may exert an antiviral effect. Additionally, its anti-inflammatory properties may also be advantageous to suppress cytokine dysregulation. Tasosartan is used to treat patients with hypertension by blocking the angiotensin II (AT1) receptor and causing vasodilation. It was already reported that tasosartan could be a potential candidate to block the interaction of viral RBD from the spike protein to the ACE2 receptor [56], thus preventing the entry of the virus into the cells. Our work shows that this compound may also prevent viral endoribonucleolytic activity, which is crucial for SARS-CoV-2 replication, being a good candidate to treat COVID-19. Dutasteride is another medication that is primarily used to treat the symptoms of an enlarged prostate. It blocks the conversion of testosterone into dihydrotestosterone, which binds to androgen receptors with a much higher affinity, leading to a poor induction of androgen receptor signaling and to a decrease in human transmembrane protease serine 2 (TMPRSS2) expression. TMPRSS2 was also shown to be necessary for the proteolytic priming of the viral S protein to allow the fusion of SARS-CoV-2 to the cellular membranes. As such, the blocking of the conversion of testosterone into dihydrotestosterone could decrease SARS-CoV-2 entry into human cells [57]. In fact, a double-blinded, placebo-controlled randomized clinical trial (EAT-DUTA AndroCoV) showed that Dutasteride has a beneficial effect in the treatment of COVID-19. Indeed, our results show that this compound has the potential to inhibit the activity of SARS-CoV-2 nsp15. This property might contribute to the reduced viral shedding observed in the trial [58]. However, we should note that Dutasteride is indicated for men and should not be used by women during pregnancy, as it can cause birth defects [59].

One of the great challenges of de novo drug design is to find compounds with desirable pharmacological properties. Therefore, the design of molecules based on scaffolds as the core structures is a way of overcoming this challenge and obtaining potential good drug candidates. Taking this into account, we consider that Meprednisone, Tasosartan and Dutasteride are excellent scaffolds, due to their effectiveness against nsp15. Two of them are FDA-approved and are being used in patients to treat other diseases, which supports their potential use as drugs. Molecular docking also revealed common features between the selected drugs, namely the interactions with nsp15 S294, Y343 and H235 amino acids, together with their ability to bind to the protein active site in the presence of a phosphate ion. Future designs of efficient drugs could ideally comprise molecules with similar scaffolds to the studied drugs in order to retain most of these features. Additionally, the discovery that sulphate groups are able to inactivate SARS-CoV-2 nsp15 reveals new features to improve or to design new scaffolds.

## 5. Conclusions

nsp15 has been demonstrating to play an important role in coronavirus pathogenesis. Moreover, there are no closely related homologues to nsp15 in human cells, which makes this ribonuclease a pertinent target for developing CoV inhibitors. nsp15 inhibition may also contribute to revert viral evasion from host cell immunity. Here, we extensively characterized the mechanism of action of SARS-CoV-2 nsp15 activity and added new and useful information for the design/development of therapeutic strategies. Additionally, effective drugs with high therapeutic potential were identified in this work. By using a repurposing approach, in which we tested only drugs that are already approved or under investigation, we may accelerate the use of these compounds as part of a strategy to fight COVID-19 in the very near future. Due to the high homology with other viruses of the family, these results can also be important to fight viruses that may emerge in the future.

## Figures and Tables

**Table 1:** Compounds predicted to bind to nsp15 ribonuclease.

Compound	Category	Binding ΔG (kcal/mol)	Chemical Formula (https://go.drugbank.com, Accessed on 5 of November 2021)
Dutasteride	Approved	−7.8	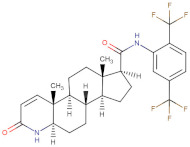
Etoposide	Approved	−7.50	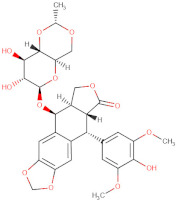
Golvatinib	Investigated	−9.2	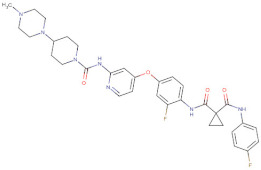
Irinotecan	Approved	−9.0	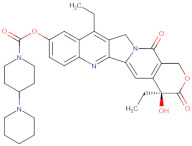
Meprednisone	Approved	−7.6	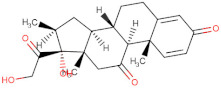
Tasosartan	Experimental	−8.3	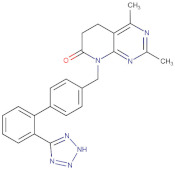

## Data Availability

Not applicable.

## Supplementary Materials

The following supporting information can be downloaded at: https://www.mdpi.com/article/10.3390/microorganisms10020342/s1, Figure S1: Chromatograms obtained during the size exclusion chromatography step of nsp15 wt and mutants. Table S1: Primers used in this study. Table S2: Plasmids used in this study.
