# Supplementary material for: The nsp15 Nuclease as a Good Target to Combat SARS-CoV-2: Mechanism of Action and Its Inactivation with FDA-Approved Drugs
Source: Microorganisms. 2022 Feb 1;10(2):342. doi: 10.3390/microorganisms10020342 (PMC8880170; doi:10.3390/microorganisms10020342)
Supplement: Supplementary file 1 [file microorganisms-10-00342-s001.zip › microorganisms-1534619-supplementary.pdf]

microorganisms

MDPI

Supplementary Material

Table S1. Primers used in this study; bases changes are underlined.

Primer Name	Sequence 5′ → 3′	Observations
T7Fw	TAATACGACTCACTATAGG	
T7Rev	GCTAGTTATTGCTCAGCGG	
Nsp15_1	GTCCGAAACAAGCTAGCCTGGCAGGTGTTACCCTG	Forward primer for the introduction of N164A mutation; creates restriction site N*he*I
Nsp15_2	CAGGGTAACACCTGCCAGGCTAGCTTGTTTCGGAC	Reverse primer for the introduction of N164A mutation; creates restriction site N*he*I
Nsp15_3	GTTATGCGTTTGAAGCGATCGTTTACGGCG	Forward primer for the introduction of H235A mutation; creates restriction site *Pvu*I
Nsp15_4	CGCCGTAAACGATCGCTTCAAACGCATAAC	Reverse primer for the introduction of H235A mutation; creates restriction site *Pvu*I
Nsp15_5	GCTGGGTGGCCTGGCACTGCTGATTGGTC	Forward primer for the introduction of H250A mutation; destroys restriction site *Bsp*MI and creates *Bts*I
Nsp15_6	GACCAATCAGCAGTGCCAGGCCACCCAGC	Reverse primer for the introduction of H250A mutation; destroys restriction site *Bsp*MI and creates *Bts*I
Nsp15_7	CCGGCAGCAGCGCATGCGTGTGCAGC	Forward primer for the introduction of K290A mutation; creates restriction site *Sph*I
Nsp15_8	GCTGCACACGCATGCGCTGCTGCCGG	Reverse primer for the introduction of K290A mutation; creates restriction site *Sph*I
Nsp15_9	GCAAATGCGTGTGCGCAGTTATCGACCTG	Forward primer for the introduction of S294A mutation; destroys restriction site *Bsg*I and creates *Fsp*I
Nsp15_10	CAGGTCGATAACTGCGCACACGCATTTGC	Reverse primer for the introduction of S294A mutation; destroys restriction site *Bsg*I and creates *Fsp*I
Nsp15_11	GCAGCCGGATCCTTATTGCAGTTTCGGATAGAAAGCTTCCACGTGACCG	Reverse primer for the introduction of T341A mutation; creates restriction site *Hind*III
Nsp15_12	GCAGCCGGATCCTTATTGCAGTTTCGGAGCGAACGTTTCCACGTGACCG	Reverse primer for the introduction of Y343A mutation; creates restriction site *Hind*III
Nsp15_13	GCAGCCGGATCCTTATTGCAGTTTCGGAGCGAAAGCTTCCACGTGACCG	Reverse primer for the introduction of T341A_Y343A mutations; creates restriction site *Hind*III

Table S2. Plasmids used in this study.

Plasmid name	Reference	Observations
pET15b_nsp15	This study	Encodes his-nsp15
pET15b_N164A	This study	Encodes his-nsp15 where N at position 164 was substituted by an alanine
pET15b_H235A	This study	Encodes his-nsp15 where H at position 235 was substituted by an alanine
pET15b_H250A	This study	Encodes his-nsp15 where H at position 250 was substituted by an alanine
pET15b_K290A	This study	Encodes his-nsp15 where K at position 290 was substituted by an alanine
pET15b_S294A	This study	Encodes his-nsp15 where S at position 294 was substituted by an alanine

pET15b_T341A	This study	Encodes his-nsp15 where T at position 341 was substituted by an alanine
pET15b_Y343A	This study	Encodes his-nsp15 where Y at position 343 was substituted by an alanine
pET15b_S294A_T341A	This study	Encodes his-nsp15 where S at position 294 and T at position 341 were substituted by alanines
pET15b_S294A_Y343A	This study	Encodes his-nsp15 where S at position 294 and Y at position 343 were substituted by alanines
pET15b_T341A_Y343A	This study	Encodes his-nsp15 where T at position 341 and Y at position 343 were substituted by alanines
pET15b_S294A_T341A_Y343A	This study	Encodes his-nsp15 where S at position 294, T at position 341 and Y at position 343 were substituted by alanines

Numbering of the amino acids according to the SARS-CoV-2 PDB 6VWW.

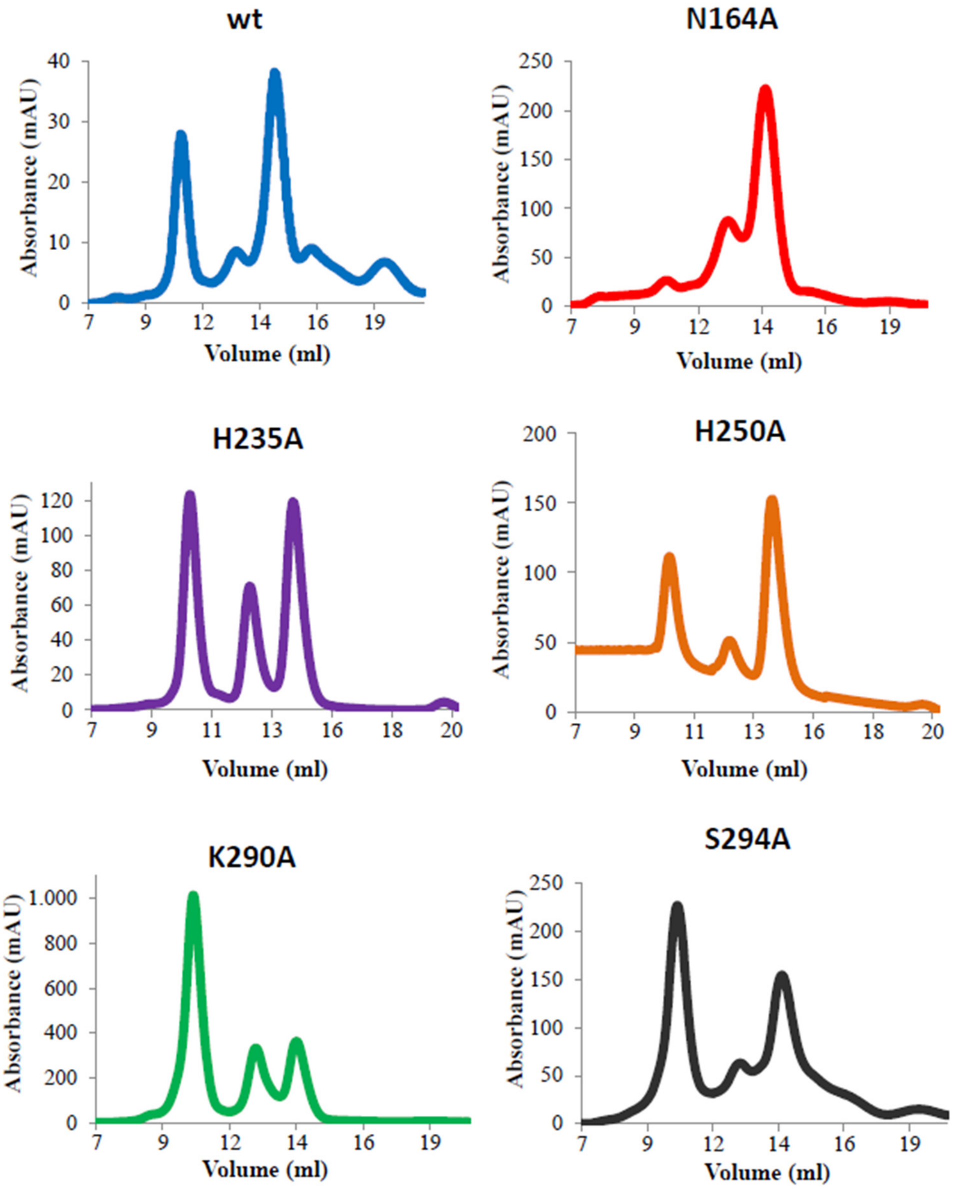

Figure S1. Chromatograms obtained during the size exclusion chromatography step of nsp15 wt and mutants, as indicate on top of the corresponding images.
